# Encouraging impacts of an Open Education Resource Degree Initiative on college students’ progress to degree

**DOI:** 10.1007/s10734-022-00817-9

**Published:** 2022-02-26

**Authors:** Rebecca Griffiths, Jessica Mislevy, Shuai Wang

**Affiliations:** 1 SRI International, Menlo Park, CA USA; 2 School of Education, Shanghai Jiao Tong University, Shanghai, 200240 China

**Keywords:** Open educational resources, Textbooks, Postsecondary education, Financing education

## Abstract

Textbooks are traditional and useful learning resources for college students, but commercial texts books have been widely criticized for their high costs, restricted access, limited flexibility, and uninspiring learning experiences. Open Education Resources (OER) are an alternative to commercial textbooks that have the potential to increase college affordability, access, and instructional quality. The current study examined how an OER degree—or pathway of OER courses that meet the requirements for a degree program—impacted students’ progress to degree at 11 US community colleges. We conducted quasi-experimental impact studies and meta-analysis examining whether OER course enrollment was associated with differences in credit accumulation and cumulative GPA over multiple terms. Overall, we found a positive effect of OER degrees on credit accumulation and no significant difference on cumulative GPA. Taken together, these results suggest students are maintaining their GPAs despite taking more courses, on average. This suggests that students taking OER courses were making faster progress towards degrees than their peers who took no OER courses.

## Introduction

Textbooks are traditional and useful learning resources for college students, but commercial texts books have been widely criticized for their high costs, restricted access, limited flexibility, and uninspiring learning experiences (Colvard et al., 2018; Hilton, 2019; Smith, 2009). These shortcomings may impact students’ academic outcomes, as many students cannot access required instructional materials or experience delays in accessing these materials, potentially leading to high withdrawal and failure rates (e.g., Colvard et al., 2018; Florida Virtual Campus, 2016). Open Education Resources (OER) have emerged as an alternative to commercial textbooks that proponents argue can lead to increased affordability, access, and quality of instruction (Colvard et al., 2018; Hilton, 2019). OER are digital materials that are freely available and openly licensed, allowing instructors and students to adapt, reuse, and share them. OER can be any type of content including entire textbooks, assessments, articles, lesson plans, videos, or individual images. Instructors can use OER in place of commercial resources to ensure that students have access to required instructional materials from the beginning of a course. Students have the opportunity to redirect these cost savings towards other educational expenses, for example, enrolling in more credits, or towards personal expenses such as food and rent. OER also has been theorized to enable improved instructional quality by prompting faculty to change the way they think about content and learning opportunities, adopting new approaches that center and empower students and nourishing a participatory culture of learning and knowledge creation (Kaatrakoski et al. 2017; Bali et al., 2020).

There has been a dramatic increase in the awareness and use of OER in recent years. A national survey of university faculty and department chairs assessing OER awareness and perceptions showed that the percentage of respondents familiar with OER increased from 34 to 46% over 3 years (Seaman & Seaman, 2018). Twenty-six percent of faculty who teach required introductory courses reported using OER for required course material in a national survey conducted in 2017/18 (Spilovoy et al., 2020). The Covid-19 pandemic may further increase the appeal of OER. For instance, the recent shift to online teaching and learning from traditional instructional settings calls for improved access to high quality digital learning resources (Means & Neisler, 2020). Additionally, the economic shock to GDP and employment has been accompanied by a sharp decline in college enrollment, particularly at community colleges, which serve a share of low-income students (Sedmak, 2020). Cost savings associated with OER may be more critical to students than ever before.

Evidence suggests that use of OER can improve students’ course outcomes (Colvard et al., 2018; Hilton, 2016, 2019). Initially, OER adoption efforts were often undertaken by individual faculty on individual courses or sections and not meaningfully connected to the broader educational goals of the institution (e.g., Hilton, 2016). A number of studies have examined course-level impacts of OER use on student outcomes, and one study examined both course completion and enrollment intensity over two terms for students who took OER courses (Fischer et al., 2015). However, previous research has not examined the effects on longer term academic outcomes of institution-level efforts that enable students to take multiple OER courses as part of a degree pathway. The impact of OER degrees may be different from unconnected course conversions, as converting entire degree programs to OER requires colleges to intentionally integrate OER work into their organizational strategies, policies, and practices, requiring coordinated efforts.

The current study examined how an OER degree pathway initiative impacted key student outcomes from studying 11 community colleges in the USA. A primary goal of the research is to determine, with as much confidence as possible, whether the availability of OER degree pathway options enables students to attempt and complete more college credits and thus progress more quickly towards attaining degrees.

### OER degree pathway

Achieving the Dream’s OER Degree Initiative aimed to increase college affordability and student success by catalyzing an institutional commitment to OER. This initiative was motivated by the expectation that an “OER degree,” or pathway of OER courses that together meet the requirements for a degree program, could set an ambitious goal for scaling up OER course offerings. Converting courses to OER could reinvigorate curriculum and pedagogy, leading to improved quality of instruction. At the same time, redesigning courses with OER is often time-consuming and demands new skills and supports for instructors. Scaling within institutions thus entailed an organizational effort that bridged departments and units, potentially enhancing institutional culture.

Launched in 2016, the OER Degree Initiative sought to promote affordability and innovation at community colleges by supporting large-scale OER adoption. Over two and a half years, the initiative supported 38 community colleges across 13 states in the USA in building degree pathways using only OER instructional materials. Eighteen of the grantees participated in multi-college consortia that received funding from the initiative. The scale of the OER Degree Initiative created an opportunity to explore how OER adoption at scale affects students, instructors, and institutions.

This initiative took place in a broader context of growing interest and support for OER programs at the state and national level, as OER have emerged as a promising and potentially transformative contribution to solving the problem of college affordability. The OER degree model was first pioneered at Tidewater Community College as a “Z degree” (indicating zero cost) and then spread across the state of Virginia and elsewhere. Subsequently, in 2018, the New York legislature allocated $16 million to the State University of New York’s (SUNY) and City University of New York (CUNY) systems to support OER work, in addition to funding the Open SUNY Textbooks (OST) program, which offers a curated selection of OER textbooks and courses at no cost through an online platform (Government of New York State, 2018). The U.S. Department of Education and many states have also launched #GoOpen initiatives to support broader adoption. In 2016, the California legislature awarded over $100 million in grants to support development of OER degrees through a “zero-textbook-costs” (ZTC) program at two dozen community colleges, and participants reported tentative though early signs of encouraging results (Burke, 2019).

Achieving the Dream’s OER Degree Initiative grantee colleges were required to convert at least one section of each of 20 courses required for specific associate degrees, though some colleges converted entire courses, and some developed more than one OER degree pathway. A requirement of the grant was that all OER degree courses be certified by Lumen Learning as meeting open license standards. The Achieving the Dream’s OER Degree Initiative required courses to use all openly licensed instructional content except in specific circumstances. For example, instructors could require students to purchase essential tangible goods, such as laboratory equipment for lab courses or art supplies for a studio course. Grantees were permitted to use copyrighted primary sources such as novels in required courses, but elective courses that used copyrighted material did not count towards the OER degree pathway. Grantees were permitted to charge fees for OER courses, but students must have unrestricted access to course materials from day 1. In many cases, colleges needed to revise courses that had been considered OER but that used proprietary content.

### Efficacy of OER

Although there was no prior study examining the effects of *OER degrees* on student outcomes systematically, the existing evidence about the impacts, potential benefits, and overall opinion of individual OER courses or sections is mostly encouraging. For instance, prior research suggests that OER courses have the potential to save students’ money with the same or modestly improved course outcomes (Fischer et al., 2015; Hilton & Laman, 2012). A synthesis study of 9 studies (Hilton, 2016) and a meta-analysis of 36 studies (Hilton, 2019) that focused on OER and its impacts in postsecondary environments found that students achieved the same or better learning outcomes when using OER compared to when they used commercial textbooks; students also saved a significant amount of money from using OER instead of commercial textbooks. The aforementioned “throughput” study authors attributed the positive impacts they detected on student course completion and enrollment intensity on access and affordability, as opposed to differences in instructional design (Fischer et al., 2015).

Additionally, in a recent meta-analysis, Clinton and Khan (2019) called for the need to provide a nuanced approach to examine academic outcomes, and they further divided student outcomes into student learning performance (e.g., GPA) and student progress towards the course completion (e.g., withdrawal rate). In the meta-analysis, they showed that there were no differences in learning performance between OER and commercial textbooks, but students using OER had a significantly lower withdrawal rate compared to students using commercial textbooks. Other research has found that broader indicators of students’ progress such as overall course completion, early credit accumulation, and cumulative college GPA are important predictors of degree attainment (Moore & Shulock, 2009).

Importantly, given that random controlled trials (RCT) testing course or program-level interventions such as OER are rarely feasible in postsecondary settings, it is critical to consider and control for student background characteristics in the study designs and analyses. For instance, Griggs and Jackson (2017) argued that researchers will not be able to generate meaningful results with external validity for OER efficacy studies without controlling for relevant variables. As an example, one confounder frequently discussed in prior studies was student prior performance (Cassidy, 2015). Clinton et al. (2019) found that students in the OER course produced higher GPA compared to students using commercial textbooks while both groups were taught and graded by the same instructor. However, students in the OER group had higher prior achievement (e.g., high-school GPA). Without controlling for student prior achievement or other confounding variables in the analyses, it is difficult to attribute the performance difference observed between the two courses to the use of OER. In addition, it is also helpful to understand whether or not OER has differential effects on subgroups of students (e.g., students with lower prior achievement) (Clinton & Khan, 2019). Thus, for the current study, the research team both controlled for background characteristics in the main impact analysis to generate accurate estimates and conducted moderation analysis to examine the differential effects of OER for subgroups.

Furthermore, Hilton (2019) indicated that, quite often, data metrics used in different studies or even within the same study can be very different, rendering it difficult for scholars to come up with synthesized results in meta-analyses. As an example, Croteau (2017) examined 3,847 college students in Georgia who used OER, but the study was based on inconsistent data (e.g., completion rates, and grade distributions) reported by different faculty members. Similarly, Ozdemir and Hendricks (2017) studied 28 faculties who provided evaluations regarding the impact of OER on student learning. Unfortunately, the outcome variables varied widely, from improved scores on exams, to anecdotal evidence, to no data or explanation at all. The lack of control of the variables used in the analysis limited the value of the overall study. Thus, the current study used the same data collection process, analysis approaches, and reporting methods across multiple settings, generating consistent results that can be synthesized.

### Research questions

In this paper, we aimed to answer two research questions:

1. Did students who took OER degree classes make greater progress towards degrees compared with similar students who take traditional classes, when we control for student background characteristics?
2. Were OER degrees more or less beneficial to particular subgroups of students (e.g., Pell grant eligible)?

## Methods

### Sample

The 38 community colleges participating in the Achieving the Dream’s OER Degree Initiative began rolling out their OER degrees as early as fall 2016, though most began offering OER degree courses in spring 2017 and continued program development through the end of 2018. Eleven of the 38 colleges in the Achieving the Dream’s OER Degree Initiative were selected to participate as “research partner colleges,” meaning they collaborated with the research team to design and conduct impact studies of their OER degrees. Criteria for selection included having capacity to meet the data requirements for the study, having conditions that would be likely to allow for strong study design, and interest in participation. Prior experience with rigorous research studies was desirable, but we also sought to include a diverse sample of colleges in terms of geography, student population, urbanicity, institutional capacity, and representation of colleges participating in consortia as well as individually (see Table 1).

**Table 1:** Characteristics of the 11 U.S. colleges that participated in the Achieving the Dream’s OER Degree Initiative as research partners

SITE	State	Locale^1^	Fall 2017 enrollment^2^	Fall 2018 Minority Serving Institution status^3^	OER Degree Consortium
College A	Virginia	City	4,000 +		Yes
College B	California	City	28,000 +	HSI	No
College C	Washington	City	12,000 +	AANAPISI	No
College D	New York	Rural	2,000 +		Yes
College E	Maryland	Suburb	22,000 +	AANAPISI	No
College F	Texas	City	40,000 +		Yes
College G	New York	Suburb	12,000 +		Yes
College H	North Carolina	City	7,000 +		No
College I	Massachusetts	City	11,000 +	AANAPISI	No
College J	Texas	City and suburb	61,000 +	HBCU, HSI	Yes
College K	New York	City	26,000 +	HSI	Yes

Minority Serving Institution status was presented as of the end of the study period. AANAPISI is Asian American Native American Pacific Islander-Serving Institutions. HBCU is Historically Black Colleges & Universities. HSI is Hispanic Serving Institutions

^1^https://nces.ed.gov/programs/maped/LocaleLookup/

^2^U.S. Department of Education, National Center for Education Statistics, Integrated Postsecondary Education Data System (IPEDS), Spring 2018, Fall Enrollment component. We rounded the enrollment numbers to the nearest thousand in efforts to maintain the anonymity of participating institutions

^3^Snyder, T.D., de Brey, C., and Dillow, S.A. (2019). Digest of Education Statistics 2018 (NCES 2020–009). National Center for Education Statistics, Institute of Education Sciences, U.S. Department of Education, Washington, DC

### Research context and procedure

The goal of the study was to test the cumulative impact of enrolling in multiple OER degree courses on students’ progress towards degree. Because the OER degree pathways took several years to develop and selection of courses to convert was based on a number of factors, including faculty interest, students generally did not have the opportunity to enroll in fully developed “pathways” of OER courses. Thus, we defined three levels of treatment for impact analyses. We considered treatment students to have received a “high dosage” of OER courses if they enrolled in three or more OER courses. We considered treatment students to have received a “low dosage” of OER courses if they enrolled in either one or two OER courses. Students in the control condition all received “no dosage” of OER courses.

As shown in Table 2, a relatively small proportion of students in the sample took three or more OER courses, ranging from 23 to 1% at several of the colleges. As much as 31 to 86% of the sample did not take any OER courses; these students served as the pool from which comparison students were identified. A total of over 21,000 students took at least 1 OER course across the 11 colleges during the course of the study.

**Table 2:** Number of students taking OER courses, by college

College	Number of OER courses taken						Total
	0	1	2	3	4	5 +	
College A	3,209	2,959	1,896	1,227	659	548	10,498
	31%	28%	18%	12%	6%	5%	100%
College B	1,129	203	46	11	4	0	1,393
	81%	15%	3%	1%	0%	0%	100%
College C	8,807	786	532	82	21	7	10,235
	86%	8%	5%	1%	0%	0%	100%
College D	2,068	490	173	52	16	6	2,805
	74%	17%	6%	2%	1%	0%	100%
College E	2,979	709	480	238	161	141	4,708
	63%	15%	10%	5%	3%	3%	100%
College F	5,039	1456	658	227	76	33	7,489
	67%	19%	9%	3%	1%	0%	100%
College G	291	41	20	13	3	0	368
	79%	11%	5%	4%	1%	0%	100%
College H	673	338	160	68	32	11	1,282
	52%	26%	12%	5%	2%	1%	100%
College I	615	596	205	76	23	9	1,524
	40%	39%	13%	5%	2%	1%	100%
College J	9,912	2,118	1,045	430	180	100	13,785
	72%	15%	8%	3%	1%	1%	100%
College K	4382	435	384	325	245	387	6158
	71%	7%	6%	5%	4%	6%	100%

**Fig. 1:**
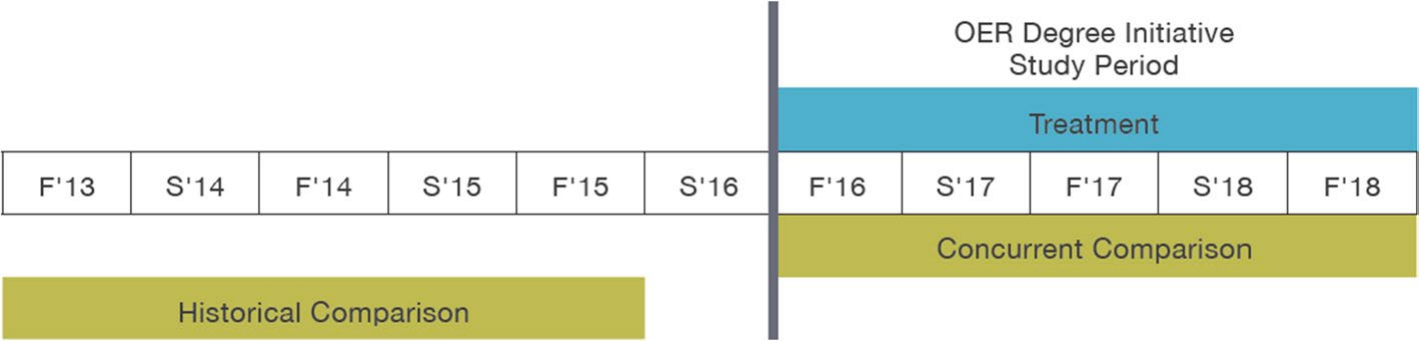
Illustrative example of concurrent versus historical comparison designs

We also worked with each research partner college to determine whether a concurrent comparison or historical comparison design would be most feasible in their institutional contexts. In the historical comparison design, we followed a cohort of students for a specified period of time following the launch of the OER Degree Initiative and compared them with a cohort of otherwise similar students (controlling for background characteristics) who enrolled before the OER degree was launched, over the same number of semesters. In the concurrent comparison design, we followed students for a specified period of time following the launch of the OER Degree Initiative, and from this pool, identified those who completed one or more OER courses as treatment students and those who took no OER course as the comparison (see Fig. 1). Several factors influenced study design choices, such as other policy changes coinciding with the launch of the OER degree or whole course conversions (which would interfere with concurrent comparisons if all students have to take OER courses). As a result, 8 of the 11 research partner colleges used a concurrent comparison design and the remaining three used a historical comparison design. The starting term and number of terms for which we followed students varied by college depending on when they rolled out OER degree courses. For some colleges with existing OER courses, we were able to follow students for as many as five fall/spring terms beginning in fall 2016. For others who required more time to develop OER courses, we were only able to follow students for as few as two or three terms, starting as late as fall 2017 or spring 2018.

### Data sources

The research team requested student-level data from the 11 research partner colleges. Specifically, we requested student demographic variables (e.g., gender), a measure of prior academic achievement such as math placement test scores, and transcript data for students enrolled in that cohort from all 11 colleges. Given that the community colleges included in our sample are broad-access institutions, many did not require students to take standardized entrance exams such as the SAT or ACT, and some had eliminated placement tests due to policy changes. In cases where such a test was not available, we created an ordinal measure of prior achievement using math course placement. We requested these data term-by-term for all students for each subsequent semester included in the impact study time period.

Specifically, we examined the impact of OER degrees on progress towards degree as measured by two student outcomes: college credits attained and cumulative college grade point average (GPA). All 11 colleges were able to provide student-level transcript data that allowed us to calculate credits accumulated over the study time frame, and 9 out of 11 colleges provided sufficient data for us to calculate cumulative GPA.

The research team conducted data screening of all datasets received by our research partner colleges to ensure they were eligible for impact analysis. Our screening criteria for conducting an impact analysis were (a) datasets contained enrollment data sufficient to identify treatment and control students; (b) datasets contained one or more outcomes of interest (or sufficient data for us to construct an outcome ourselves); and (c) datasets contained sufficient linking data to allow for following each individual student over a sequence of semesters. Additionally, we cross-checked all datasets with our site-specific study implementation plans to confirm the datasets contained the appropriate student populations, enrollment terms, courses, and programs of study. We worked with each individual college to clarify any issues regarding missing data, data linking, and data coding.

The research team also conducted an online survey of instructors at all 38 colleges in 2018. In total, 906 instructors participated in the 2018 survey (a 65% response rate). The research collected information regarding instructors’ experiences teaching with OER, including availability of training (“Have you received any training or professional development services related to the OER degree pathway?” where 1 = Yes and 0 = No); reported leadership support (“To what extend has lack of administrative support been a barrier in developing OER degree courses?” with response options from 1 = Not a barrier to 4 = Major barrier and lower values indicative of more leadership support); perceptions of sustainability (“Do you see OER degree pathways as a long-term option for your institution?” with response options from 1 = Definitely to 4 = Definitely not); propensity for scale (“How likely are you to recommend OER to a friend or colleague teaching the same course you do?” with response options from 0 = Extremely unlikely to 10 = Extremely likely); and share of adjunct instructors (“What if your current employment status?” where 1 = Adjunct/part time instructor and 0 = Other). The average across instructors’ responses to these questions for each institution was included as predictors in the meta-analyses for the current study.

Additionally, during the data cleaning process, we applied consistent rules for variable coding to standardize variables across datasets. For example, different colleges reported different data regarding students’ Pell status. Some college partners reported Pell recipient whereas some reported Pell eligibility, and some reported a single datapoint for each student whereas other partners reported term-specific Pell information. We coded students as “Yes” for Pell status if institutions recorded them as being either Pell recipient or Pell-eligible for any enrollment term during the applicable eligibility window given the study design. We also coded students as “Yes” for underrepresented minority student if they racially/ethnically identified as “African American/Black, American Indian/Alaska Native, Hispanic, Pacific Islander/Hawaiian Native, Two or more races/ethnicities, or Other.” We also collected student ages and their starting terms at the institutions.

### Analysis

We conducted a series of quasi-experimental impact analyses for all 11 colleges to examine the extent to which enrollment in OER courses was associated with improved student outcomes as measured by college credits attained and cumulative college GPA.

Prior to analysis, we checked all cleaned datasets for baseline differences for students’ prior achievement and demographic variables in accordance with What Works Clearinghouse™ standards handbook (Department of Education, 2020). We considered our treatment and control analytic samples to be non-equivalent if the effect size of any baseline difference was greater than 0.25. For continuous variables, we calculated Hedges’ *g* as the effect size, and for categorical variables, we calculated Cohen’s *d* as the effect size. When the baseline difference (e.g., student prior achievement, gender, and age) between the treatment and control samples had an effect size greater than 0.25, we conducted propensity score matching (PSM) for impact analyses to produce a matched sample statistically equivalent at baseline. PSM is a statistical matching technique that creates artificially similar analytic samples in an attempt to reduce possible statistical bias from observed variables. We employed nearest neighbor matching and used *MatchIt* in R for PSM, and allowed treatment students to match with multiple control students, and we allowed for replacement (Ho et al., 2011). We calculated PSM weights to account for the number of times a given control student was matched with treatment students. We applied PSM weights to all subsequent baseline checks and impact analyses. After the matching procedure was completed, we again conducted baseline checks on the matched samples. If statistical equivalence was not achieved, we conducted another round of PSM. If statistical baseline was achieved, we proceeded with our impact analysis.

In the first stage of our analysis, we used ordinary least squares linear regression (OLS) for conducting our impact analyses for each of the institution. We included students’ prior achievement (including GPA and placement scores), demographic variables (including Pell Status, Gender, and Ethnicity), starting term at the institution, and number of semesters enrolled as statistical controls in our models. We opted for a conservative approach of controlling for the number of semesters in which students were enrolled to the risk that students received the treatment as a result of longer retention (giving them the opportunity to take more OER courses). We report treatment effect sizes as Hedges’ *g* when treatment effects are statistically significant. We also conducted subgroup analyses for low-income students (i.e., students coded as “Yes” on Pell status) and for underrepresented minority students by adding interaction effects to the model.

In the second stage of our analysis, we used a meta-analytic approach to summarize findings across institutions (Cooper & Hedges, 1994; Lipsey & Wilson, 2001). Although the impact studies for each institution followed a generally consistent approach, there was some variation among the OER initiatives and the institutions rolling them out as described (see the “Research context and procedure” section). Using meta-analysis offered more flexibility to combine the results across individual studies with variation as compared to combining the data into a single analysis. Though it was not possible to test for interactions between student- and institutional-level characteristics, our meta-analytic approach allowed us to explore implementation and study design features that are associated with the size of the impact in different institutions.

## Results

### Descriptive statistics

Table 3 presents the characteristics of the final analysis samples for each site in terms of size and student demographics. These data are for the matched samples, after propensity score matching was used to establish baseline equivalence between the treatment and comparison conditions. The sample size available for analysis ranged considerably across the 11 colleges, with some having samples in the low hundreds and others approaching 5,000. With the exception of one site, the average student age was around 20 years old. At least a third or more of the sample at each college consisted of students eligible for or who had received Pell grants. The proportion of students from historically underrepresented ethnic minority groups ranged considerably across colleges from 25 to 88%.

**Table 3:** Demographic characteristics of the final analysis samples, by college

College	Student characteristics	Control (0 OER courses)			Low (1–2 OER courses)			High (3 + OER courses)		
		*N*	M	SD	*N*	M	SD	*N*	M	SD
College A	URM	2,068	0.78	0.41				2,191	0.81	0.39
	Pell status	2,068	0.59	0.49				2,191	0.63	0.48
College B	URM	420	0.65	0.48	152	0.67	0.47			
	Pell status	na	na	na	na	na	na			
College C	URM	306	0.70	0.46				65	0.72	0.45
	Pell status	306	0.76	0.43				65	0.77	0.42
College D	URM	1048	0.89	0.31				574	0.89	0.31
	Pell status	1048	0.38	0.49				574	0.38	0.49
College E	URM	280	0.25	0.44				161	0.27	0.44
	Pell status	280	0.45	0.50				161	0.49	0.50
College F	URM	565	0.37	0.48				140	0.36	0.48
	Pell status	565	0.40	0.49				140	0.39	0.49
College G	URM	93	0.25	0.44	59	0.25	0.44			
	Pell status	93	0.59	0.50	59	0.56	0.50			
College H	URM	171	0.70	0.46				76	0.64	0.48
	Pell status	171	0.48	0.50				76	0.47	0.50
College I	URM	168	0.35	0.48	192	0.31	0.46			
	Pell status	168	0.34	0.47	192	0.32	0.47			
College J	URM	2993	0.88	0.33	2141	0.84	0.36			
	Pell status	2993	0.51	0.50	2141	0.51	0.50			
College K	URM	1289	0.38	0.48	681	0.38	0.48			
	Pell status	1289	0.69	0.46	681	0.67	0.47			

M indicates “mean” or average, which refers to the proportion of students who were URM or received Pell grants. SD indicates standard deviation, a measure of the variation within a group. For each college, we analyzed results for students who received high dosage where sample size permitted; otherwise, we analyzed results for students who received low dosage of OER courses

### RQ1 main impact analysis

#### Credit accumulation

In 6 of the 11 colleges, treatment students taking OER courses accumulated significantly more course credits than those who had not taken any OER courses (see Table 4). The number of additional credits accumulated by treatment students ranged from 2 to 8 across these six schools. In the remaining 5 colleges, students’ credit accumulation in the two conditions was not statistically different after controlling for differences in student characteristics.

**Table 4:** Credit accumulation results, by college

Site	Dosage	Credit	SE	*p* value	ES
College A	High	3.14	0.39	< 0.0001***	0.23
College B	Low	1.88	0.94	0.046*	0.12
College C	High	1.75	1.61	0.28	0.11
College D	Low	2.44	0.69	< 0.0001***	0.14
College E	High	7.90	0.94	< 0.001***	0.56
College F	High	− 3.37	2.30	0.14	− 0.13
College G	Low	1.26	4.07	0.758	0.05
College H	High	7.30	1.50	< 0.001***	0.51
College I	Low	0.13	1.33	0.92	0.01
College J	Low	5.16	0.43	< 0.001***	0.27
College K	High	1.05	0.82	0.201	0.05

*** denotes *p* < 0.001. * denotes *p* < 0.05

Credit is impact estimate from 11 separate weighted regression models when student prior achievement and background characteristics are controlled for

Table 4 also shows the estimated effect size for each of the research partner colleges on credit accumulation. An effect size is a standardized measure of the magnitude of an effect and is calculated as the standardized mean difference between two groups. The effect size increases in magnitude as the difference between the groups on the outcome measure gets larger. The U.S. Department of Education’s What Works Clearinghouse (2017) defines effect sizes equal or greater than 0.25 as substantively important, regardless of their statistical significance. The estimated effects for 3 of the 11 colleges exceed this threshold (Colleges E, H, and J), and a fourth approaches it (College A).

**Fig. 2:**
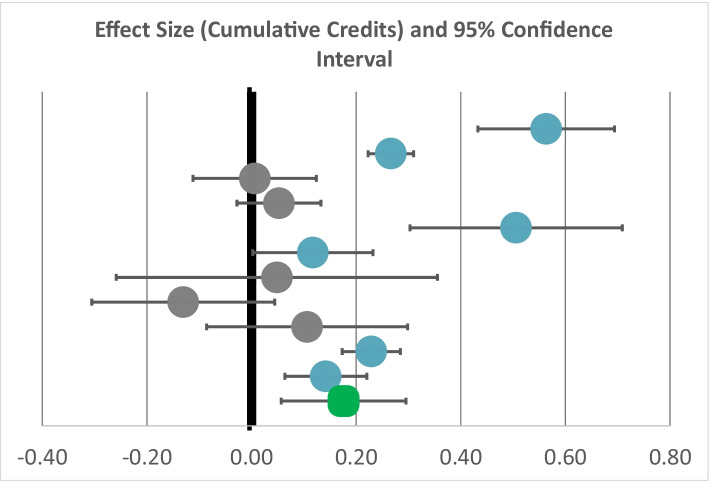
Estimated impact on cumulative credits, by institution. Each circle represents the estimated impact of OER degrees on cumulative credits in terms of effect sizes (Hedge’s *g*). The length of the horizontal line through the circle represents the 95% confidence interval around the impact estimate. The longer the line, the more uncertainty there is around the true impact of the OER degree. The degree of uncertainty is influenced by several factors, including but not limited to the size of the sample used to produce the estimate. The green box at the bottom of the graph displays the grand mean and confidence interval of the included studies. Significant positive findings (blue circles) to the right of the bolded 0.00 line indicate that the number of credits accumulated was higher for students taking OER courses, while gray circles indicate no significant differences of credit accumulation between the treatment and comparison students

The forest plot in Fig. 2 presents individual estimates and their confidence intervals for each site as well as the aggregate estimate across sites. A confidence interval that crosses the 0.00 line indicates that the estimated impact was not statistically significant from zero, which is the case for 5 of the 11 colleges. The estimated impacts for the remaining six colleges were positive and statistically significant. Across the 11 colleges, we found a small positive effect (Hedge’s *g* = 0.18) on credit accumulation.

Figure 2 also reveals that there was considerable variability across the colleges, suggesting the need to look more deeply into the conditions and practices associated with more and less favorable outcomes for OER degree implementations. To further explore the circumstances under which OER degrees were effective, we coded each impact study for the conditions under which the OER degree was implemented. These features included institution size, the share of instructors reporting that they volunteered to teach OER courses, adjuncts, reported leadership support, and availability of training. In addition, we tested whether study design features were associated with the effect size, including the type of comparison group, the number of semesters included in study time period, and whether we controlled for the number of semesters. Across colleges, none of these variables was found to be significantly related to OER degree impacts on cumulative credits.

#### Students’ cumulative GPA

Nine of the 11 colleges provided sufficient data to calculate students’ cumulative GPA. Of those, treatment students taking OER courses achieved a significantly higher GPA than those who had not taken any OER courses at just one college; results approached statistical significance at a second. The results were negative and statistically significant in two others, such that control students had a higher GPA than students taking OER courses, on average, by about half a letter grade. In the remaining 5 colleges, students’ cumulative GPA in the two conditions was not statistically different after controlling for differences in student characteristics (see Table 5).

**Table 5:** Cumulate GPA results by college

Site	Dosage	GPA	SE	*p* value^a^	ES
College A	High	NA	NA	NA	NA
College B	Low	0.052	0.10	0.589	0.050
College C	High	− 0.029	0.13	0.830	− 0.029
College D	Low	NA	NA	NA	NA
College E	High	0.000	0.12	1.000	0.000
College F	High	− 0.157	0.08	0.048*	− 0.175
College G	Low	0.159	0.11	0.164	0.223
College H	High	0.214	0.11	0.060	0.239
College I	Low	− 0.437	0.11	< 0.001***	− 0.398
College J	Low	0.187	0.03	< 0.001***	0.191
College K	High	0.014	0.04	0.731	0.015

*** denotes *p* < 0.001. * denotes *p* < 0.05

GPA is impact estimate from 9 separate weighted regression models when student prior achievement and background characteristics are controlled for

**Fig. 3:**
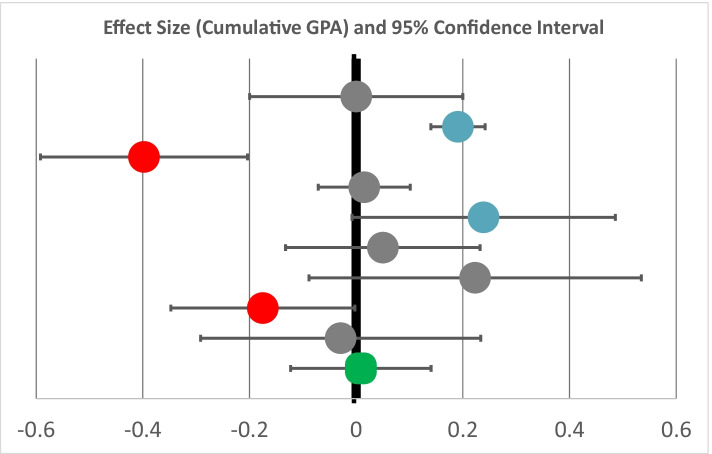
Estimated impact on cumulative GPA, by institution. Each circle represents the estimated impact of OER degrees on cumulative GPA in terms of effect sizes (Hedge’s *g*). The length of the horizontal line through the circle represents the 95% confidence interval around the impact estimate. The longer the line, the more uncertainty there is around the true impact of the OER degree. The degree of uncertainty is influenced by several factors, including but not limited to the size of the sample used to produce the estimate. The green box at the bottom of the graph displays the grand mean and confidence interval of the included studies. Significant positive findings (blue circles) to the right of the bolded 0.00 line indicate that cumulative GPA was higher for students taking OER courses, while red circles to the left indicate that cumulative GPA was lower for students taking OER courses. Gray circles indicate no significant differences of cumulative GPA between the treatment and comparison students

Figure 3 shows the estimated effect size for each of the research partner colleges on cumulative GPA. The overall effect size for the estimated impact on cumulative GPA across the nine sites was near zero and not statistically significant (Hedge’s *g* = 0.01). With statistically significant results for only three institutions, and cumulative GPA as an available outcome for only 9 of the 11 colleges, we did not explore potential factors associated with more and less favorable outcomes.

While our study found little to no impact of taking OER courses on students’ cumulative GPA, coupled with the positive findings for credit accumulation, these results suggest that students are maintaining their GPAs despite taking more courses, on average.

### RQ2: analysis of effects by subgroup

For sites with significant impacts overall, we conducted subgroup analyses to explore the extent to which enrolling in OER courses may have different impacts on (a) underrepresented minority (URM) students and (b) Pell-eligible and/or Pell-receiving students. Table 6 provides the results of the subgroup impact analyses.

**Table 6:** Estimated impact of OER degrees on cumulative credits and GPA, overall and by subgroup

Site	Cumulative credits					Cumulative GPA				
	Overall	Pell	Non-Pell	URM	Non-URM	Overall	Pell	Non-Pell	URM	Non-URM
College A	3.14	—	—	2.82	4.40	Na	Na	Na	Na	Na
College B	1.88	—	—	—	—	—	—	—	—	—
College C	—	—	—	—	—	—	—	—	—	—
College D	2.44	3.99	1.37	—	—	Na	Na	Na	Na	Na
College E	7.90	5.12	10.19	—	—	—	—	—	—	—
College F	—	—	—	—	—	− 0.16	—	—	—	—
College G	—	—	—	—	—	—	—	—	—	—
College H	7.30	—	—	—	—	—	—	—	—	—
College I	—	—	—	—	—	− 0.44	—	—	—	—
College J	5.16	6.89	3.38	—	—	0.19	0.30	0.07	—	—
College K	—	—	—	—	—	—	—	—	—	—

— denotes not significant; *Na*, not available

In three of the six sites with statistically significant impact on credit accumulation overall, this difference also appears to be associated with Pell status. For two of the colleges, Pell students appear to have benefitted even more. In other words, the number of credits earned by Pell students taking OER courses relative to their Pell-eligible peers was significantly higher than the number of credits earned by non-Pell students taking OER courses relative to their non-Pell-eligible peers. In one site with statistically significant difference in credit accumulation, non-URM students benefitted more than URM students.

In the two sites with statistically significant impact on cumulative GPA, this difference was positively associated with Pell status for one of the sites. Here, Pell-eligible students enrolled in one or two OER courses earned GPAs of 0.30 points greater than otherwise similar students whereas non-Pell-eligible students enrolled in one or two OER courses had GPAs 0.07 points greater than otherwise similar students. Racial/ethnic identity was not associated with whether OER course enrollment affected cumulative GPA for either of the sites.

Our observation of few significant interaction effects means that OER degree programs were generally benefitting different groups of students to a similar degree, and in a few cases, Pell students may have benefitted more.

## Discussion

A primary goal of the study was to determine, with as much confidence possible, whether the availability of OER degree options enabled students to progress more quickly towards attaining degrees and whether effects varied for different types of students. The research team worked with 11 colleges to conduct quasi-experimental impact studies examining whether OER course enrollment was associated with differences in credit accumulation and cumulative GPA over multiple terms.

Ultimately, the positive associations between OER course taking and credit accumulation observed in this study are encouraging. In 6 of the 11 colleges, treatment students taking a mix of high and low dosage of OER courses earned significantly more course credits than those who had not taken any OER courses. This finding suggests that students taking OER courses were making faster progress towards degrees than their peers who took no OER courses. Our findings are consistent with prior research regarding the impact of OER on indicators of student progress towards graduation. Fischer et al. (2015) also found that students in courses using OER enrolled in a significantly higher number of credits the next semester. Additionally, a recent meta-analysis (Clinton et al. 2019) on OER studies found a significant reduction in the likelihood of students who withdrew from OER courses, which would also accordingly accumulate more credits towards their degrees.

At the same time, the examination of an OER degree’s impact on students’ cumulative GPA yielded mixed results across colleges, and a nonsignificant finding overall. In reviewing 9 OER efficacy studies, Hilton (2016) found that 1 of the 9 studies showed that OER usage led to worse learning outcomes in most instances; three studies reported significant learning gains associated with the use of OER; three studies did not find significant differences between the OER and non-OER groups; and two studies did not report the significance tests. Similarly, in the Clinton et al. (2019) meta-analysis, the authors did not find that students in courses with OER are outperforming or underperforming their peers in non-OER courses in terms of course grades or exam scores. Taken together, these studies suggest that neither individual OER courses nor OER degree pathways supported better or worse learning performance. Importantly, while our study found little to no impact of taking OER courses on students’ cumulative GPA, coupled with the positive findings for credit accumulation, these results suggest that students are maintaining their GPAs despite taking more courses, on average.

### Limitations

One challenge we encountered in this study was that colleges generally did not offer OER courses to students as explicit pathways. Accordingly, students tended to enroll in OER courses on an ad hoc basis, most students enrolled in a mix of OER and non-OER courses, and few students enrolled in four or more OER courses during the study period. This ultimately limited our ability to test the cumulative impact of higher dosage of OER courses.

Additionally, as a quasi-experimental study, our analyses could only account for factors for which we could obtain data, such as prior test scores and student demographics. Even after accounting for these factors, there could be other differences between students and instructors in the OER and control conditions that this study could not address. For example, the study did not include data on student characteristics such as self-discipline and time management skills. Therefore, the results reported in this study may be attributable, in part, to these unmeasured factors.

Lastly, we opted for a conservative approach of controlling for the number of semesters in which students were enrolled in the academic impact analyses. This approach reduces the risk that students received the treatment as a result of longer retention (giving them the opportunity to take more OER courses), but conversely raises a risk that the benefits of OER course taking are underestimated, given that OER use is theorized to positively affect retention.

### Future directions

Future research can shed additional light on what factors of an OER degree made a difference for students’ credit accumulation—be it affordability, access, instructional quality, or some combination. Looking ahead, colleges should consider whether OER programs might best be organized around degree pathways or some other objective, such as maximizing impact in large enrollment courses. Those that are committed to degree pathways should explore ways to ensure that students can actually experience these courses as pathways, for example, through mechanisms such as block scheduling and advising. Future studies could examine the full effect of OER degrees once such pathways are fully implemented.

## Conclusion

Despite these challenges, the current study was the first to examine the impacts of OER degrees on progression of students towards degree across multiple semesters. This study was also the first to apply consistent design principles, including controlling for student characteristics, and outcomes measures for impact studies across multiple institutions, supporting a strong meta-analysis approach. Overall, we found encouraging results supporting continued application and study of these programs. The approach of examining the impact of program-level interventions on student progress towards degrees provides an alternative model to studies of course-level impacts, as this approach can shed light on how programs which are theorized to have cumulative benefits with increased exposure affect students’ degree attainment.
